# Long-Term Evaluation of Roux-en-Y Hepaticojejunostomy in Benign Biliary Diseases: A Retrospective Single-Center Study

**DOI:** 10.7759/cureus.103525

**Published:** 2026-02-13

**Authors:** Pravesh Mathur, Sadaf Ali Bangri, Mohd Reyaaz Lattoo, Shradha Saxena

**Affiliations:** 1 Department of Surgical Gastroenterology, All India Institute of Medical Sciences, Bhopal, Bhopal, IND; 2 Department of Surgical Gastroenterology, Sher-i-Kashmir Institute of Medical Sciences (SKIMS), Srinagar, IND; 3 Department of Pathology, Chirayu Medical College and Hospital, Bhopal, IND

**Keywords:** bile duct, biliary, cholangitis, complications, hepaticojejunostomy

## Abstract

Introduction: Roux-en-Y hepaticojejunostomy is the standard procedure for restoring biliary-enteric continuity in benign biliary diseases, including choledochal cysts, post-cholecystectomy bile duct injuries, oriental cholangiohepatitis, and benign strictures. This retrospective study aimed to evaluate the long-term outcomes of Roux-en-Y hepaticojejunostomy performed for various benign biliary diseases, with particular emphasis on the incidence, timing, risk factors, and management of late complications, primarily anastomotic stricture and recurrent cholangitis.

Materials and Methods: This single-center retrospective study analyzed 100 consecutive patients who underwent Roux-en-Y hepaticojejunostomy for benign biliary disease between January 2015 and March 2022. Data were retrieved from a prospectively maintained database with a minimum six-month follow-up. The median duration of follow-up was nine months (range: 6-15 months). Inclusion criteria included patients of any age with benign indications; exclusions included malignancy, major comorbidities, and referrals from other centers. Standard technique involved end-to-side anastomosis using 4-0/5-0 absorbable sutures, often via the Hepp-Couinaud approach. Statistical analysis was performed using the chi-square test.

Results: The cohort comprised 100 patients, predominantly 66 (66%) female patients, and most commonly aged 31-40 years. The primary indication for Roux-en-Y hepaticojejunostomy was choledochal cysts in 52 (52%) patients, followed by bile duct injuries in 33 (33%) patients. Long-term complications included recurrent cholangitis in 39 (39%) patients and anastomotic stricture in 20 (20%) patients. Stricture rates were the highest among patients with bile duct injuries. There was no significant association between sex and stricture development (p = 0.58). A near-significant trend was observed between younger age (<40 years) and a higher stricture incidence (p = 0.063). Postoperative bile leakage (p = 0.021) and cholangitis (p < 0.001) were strong predictors of anastomotic stricture formation.

Conclusion: Roux-en-Y hepaticojejunostomy for benign biliary diseases is associated with considerable late morbidity, particularly following bile duct injury. Postoperative bile leak and early cholangitis emerged as key modifiable predictors of anastomotic stricture. From a technical perspective, construction of a wide, tension-free mucosa-to-mucosa anastomosis at the hepatic hilum, preferably using the Hepp-Couinaud approach whenever feasible, is likely protective by ensuring optimal vascularity and reducing anastomotic narrowing. Younger patients may therefore require closer surveillance.

## Introduction

Hepaticojejunostomy, most commonly performed as a Roux-en-Y procedure, serves as a cornerstone of hepatobiliary surgery for restoring biliary-enteric continuity in patients with benign biliary conditions. These include iatrogenic bile duct injuries (often following cholecystectomy), choledochal cysts, oriental cholangiohepatitis (recurrent pyogenic cholangitis), and benign biliary strictures [1]. The technique involves excision of diseased biliary segments and anastomosis to a Roux-en-Y jejunal limb, effectively addressing obstruction and preventing recurrent complications, such as cholangitis, hepatolithiasis, and progression to secondary biliary cirrhosis [1,2].

Although short-term outcomes have markedly improved in specialized centers, with operative mortality approaching zero, long-term morbidity persists as a significant challenge. Anastomotic stricture at the hepaticojejunostomy site remains one of the most concerning late complications, often leading to recurrent ascending cholangitis, jaundice, intrahepatic stone formation, liver abscesses, and secondary biliary cirrhosis in advanced untreated cases [3,4]. Strictures typically manifest within the first few years after surgery, although delays of several years are possible, necessitating prolonged surveillance.

Risk factors for stricture development include concomitant vascular injury, postoperative bile leak, repair by non-specialist surgeons, and certain etiologies or timing of intervention (such as intermediate repair in bile duct injuries). Advances in interventional techniques, including percutaneous balloon dilatation and endoscopic management, offer effective nonsurgical options in many cases and achieve high success rates. However, some patients still require revision surgery, highlighting the need for meticulous techniques, risk stratification, and vigilant follow-up [3-6]. Despite these insights, data on extended follow-up across diverse benign etiologies remain limited, with few studies addressing combined clinical, radiological, and laboratory monitoring for early detection.

Although hepaticojejunostomy is well established, there is limited contemporary data from India examining long-term outcomes across different benign etiologies, particularly bile duct injury, within the local socio-demographic and referral context. Variations in disease spectrum, timing of referral, and access to specialist hepatobiliary care may influence outcomes in this setting. The study was therefore designed not only to evaluate long-term outcomes of Roux-en-Y hepaticojejunostomy but also to compare complication patterns between major etiological groups, especially bile duct injury and choledochal cysts, which emerged as a key finding in our results.

This retrospective study aimed to evaluate the long-term outcomes of Roux-en-Y hepaticojejunostomy performed for benign biliary diseases, with particular emphasis on the incidence, timing, risk factors, and management of late complications such as anastomotic strictures and recurrent cholangitis. The specific objectives were to determine the prevalence and patterns of long-term complications, including anastomotic strictures, recurrent cholangitis, hepatolithiasis, liver abscesses, and secondary biliary cirrhosis; to identify potential risk factors associated with anastomotic stricture development, such as postoperative bile leak, recurrent cholangitis episodes, timing of bile duct injury repair, and underlying etiology; to assess the effectiveness of diagnostic approaches involving clinical, laboratory, and radiological parameters, as well as the outcomes of interventional management strategies, including percutaneous, endoscopic, and surgical interventions; and to emphasize the value of extended follow-up protocols in improving patient quality of life and preventing progression to severe sequelae.

## Materials and methods

Study design and ethical considerations

This study was designed as a retrospective analysis with partial prospective data collection conducted in the Department of Surgical Gastroenterology at Sher-i-Kashmir Institute of Medical Sciences (SKIMS), Srinagar, Jammu, and Kashmir, India. Prior ethical approval was obtained from the Institutional Ethical Committee of SKIMS (SIMS 131/IEC-SKIMS/2022-147, dated 26-04-2022). The study was performed in accordance with the ethical principles outlined in the Declaration of Helsinki (1964) [7] and its subsequent amendments. All patient data were handled with strict confidentiality, and only anonymized information was used for the analysis.

Study period and patient selection

This retrospective study included the records of all consecutive patients who underwent Roux-en-Y hepaticojejunostomy for benign biliary diseases between January 2015 and March 2022. Patient information was retrieved from a prospectively maintained departmental database that included retrospective case entries, supplemented by 18 months of concurrent prospective documentation. The sample size was calculated a priori using the G*Power software (version 3.1.9.2; Heinrich Heine University, Düsseldorf, Germany). Based on a reported prevalence rate of 25% for post-hepaticojejunostomy cholangitis and an estimated effect size of 0.15 [8], a minimum of 96 patients were required to achieve 95% statistical power with a 5% alpha error. The final sample size was increased to 100 to account for potential data attrition and facilitate group allocation. Thus, 100 patients who fulfilled the predefined inclusion and exclusion criteria were included in the final analysis. All patients had a minimum postoperative follow-up period of six months.

Inclusion and exclusion criteria

Patients of any age who underwent Roux-en-Y hepaticojejunostomy for benign conditions (including choledochal cysts, post-cholecystectomy bile duct injuries, oriental cholangiohepatitis, and other benign biliary strictures) were considered eligible. Only mentally sound individuals who were capable of providing informed consent for follow-up and data utilization were included. Patients were excluded if they were within six months of surgery, had significant mental illness, had multiple major comorbidities that would confound long-term assessment, were referred from other centers where the primary surgery was not performed by a surgeon at our institution, or had any malignant biliary pathology. The patients with American Society of Anesthesiologists (ASA) Physical Status class IV or higher, reflecting severe systemic disease posing a constant threat to life, were excluded.

Surgical technique

**Figure 1:**
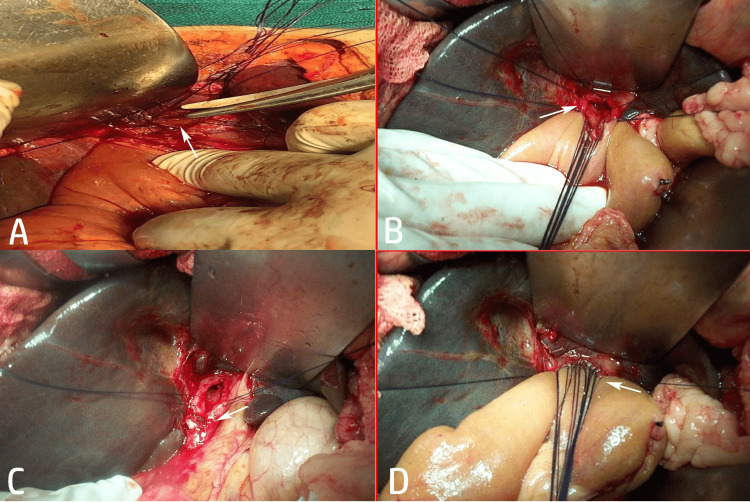
Intraoperative steps of Roux-en-Y hepaticojejunostomy in benign biliary disease. (A) Intraoperative view showing the transected bile duct at the hepatic hilum (arrow) following bile duct injury; (B) placement of sutures for the posterior layer of the hepaticojejunostomy, with the arrow indicating the biliary stump prepared for anastomosis; (C) completed posterior layer of the hepaticojejunostomy anastomosis, with the arrow highlighting the bile duct lumen and mucosa-to-mucosa approximation; (D) completion of the anterior layer of the Roux-en-Y hepaticojejunostomy, with the arrow demonstrating the final tension-free biliary–enteric anastomosis. Original images of a patient from the study, used with patient's permission.

All hepaticojejunostomies were performed using the standard Roux-en-Y technique with a 40-60 cm Roux limb. The biliary-enteric anastomosis was constructed in an end-to-side fashion, usually at or near the hepatic hilum (Hepp-Couinaud approach, whenever feasible), employing interrupted or continuous 4-0 or 5-0 absorbable sutures. In cases of bile duct injury, the timing of repair was categorized as early (<72 h), intermediate (3-21 days), or late (>21 days), depending on the referral pattern and clinical presentation (Figure 1). Selective access loop creation was performed in patients diagnosed with oriental cholangiohepatitis.

Follow-up protocol and complication definitions

A standardized institutional follow-up protocol was implemented, with patients advised to attend the outpatient department every three months for the first five postoperative years. Routine clinical evaluation included a detailed history, physical examination, liver function tests (with special attention to alkaline phosphatase and direct bilirubin), and abdominal ultrasonography. Patients presenting with fever, jaundice, or abdominal pain underwent urgent laboratory investigation and radiological imaging. Long-term complications were defined as those occurring six months or more after surgery. Ascending cholangitis was diagnosed clinically based on the triad of fever (with or without chills/rigors), abdominal pain, and deranged liver function test results. Hepaticojejunostomy anastomotic stricture was definitively confirmed by magnetic resonance cholangiopancreatography (MRCP), demonstrating narrowing at the anastomosis site with proximal intrahepatic biliary dilatation. Hepatolithiasis, liver abscess, and secondary biliary cirrhosis were diagnosed using a combination of clinical, laboratory, and imaging criteria (including ultrasonography, MRCP, and transient elastography where indicated).

Data collection and variables recorded

Comprehensive data were collected using a predesigned structured format. Recorded variables included demographic details (age and sex), primary indication for hepaticojejunostomy, intraoperative findings, surgical technique details, early postoperative complications, timing and nature of delayed complications, management strategies employed (conservative, percutaneous, endoscopic, or surgical), duration of follow-up, and final clinical outcomes. The median duration of follow-up was nine months (range: 6-15 months). 

Statistical analysis

All statistical analyses were performed using the Statistical Package for Social Sciences (SPSS) software (version 30.0; IBM Corp., Armonk, NY). Demographic data are presented as frequencies and percentages. The association between categorical variables was analyzed using the chi-square test. Statistical significance was set at p < 0.05.

## Results

The study cohort of 100 patients who underwent hepaticojejunostomy was predominantly female and most commonly aged 31-40 years. The primary surgical indication was choledochal cyst in 52 (52%) patients, followed by bile duct injury in 33 (33%) patients. Postoperative complications occurred in a significant proportion of patients, with cholangitis in 39 (39%) patients and hepaticojejunostomy stricture in 20 (20%) patients being the most frequent complications. This profile indicates that the procedure is often performed for benign biliary diseases in young to middle-aged adults, with inflammatory and stricturing complications representing the principal challenges for long-term success. The female predominance aligns with the higher incidence of conditions such as choledochal cysts in this demographic group (Table 1).

**Table 1:** Demographic and clinical characteristics of the study cohort (N = 100). Data are expressed as number (percentage). *Values exceed 100% as some patients experienced more than one complication. Overall, 39 patients (39%) developed at least one postoperative complication.

Parameter	Category	n	%
Sex	Male	34	34
	Female	66	66
Age group (years)	10-20	9	9
	21-30	27	27
	31-40	34	34
	More than 40	30	30
Primary indication for hepaticojejunostomy	Choledochal cysts	52	52
	Bile duct injuries	33	33
	Others	15	15
Postoperative complications*	Cholangitis	39	39
	Hepaticojejunostomy stricture	20	20
	Hepatolithiasis	7	7
	Liver abscess	3	3
	Small bowel obstruction	1	1
	Secondary biliary cirrhosis	5	5

Analysis of complications by surgical indication revealed significant variations. Patients who underwent hepaticojejunostomy for bile duct injury repair had the highest incidence of both cholangitis and hepaticojejunostomy strictures. The "other" indications group demonstrated an intermediate risk profile. In contrast, patients with choledochal cysts had comparatively lower rates of major complications. The inference is that the underlying pathology and surgical context, particularly the presence of a prior traumatic or iatrogenic injury, profoundly influence postoperative outcomes. This stratification of risk is crucial for preoperative counseling and for tailoring the intensity of postoperative surveillance, with bile duct injury patients requiring the most vigilance (Table 2).

**Table 2:** Distribution of postoperative complications according to primary indication for surgery. Values are presented as number (percentage within each indication group).

Complication	Choledochal cyst (n = 52)	Bile duct injury repair (n = 33)	Others (n = 15)	Total (N = 100)
Hepaticojejunostomy stricture	6 (11.5%)	11 (33.3%)	3 (20.0%)	20 (20.0%)
Cholangitis	11 (21.2%)	24 (72.7%)	4 (26.6%)	39 (39.0%)
Hepatolithiasis	2 (3.8%)	3 (9.1%)	2 (13.3%)	7 (7.0%)
Liver abscess	0 (0%)	3 (9.1%)	0 (0%)	3 (3.0%)
Small bowel obstruction	0 (0%)	0 (0%)	1 (6.6%)	1 (1.0%)
Secondary biliary cirrhosis	1 (1.9%)	2 (6.1%)	2 (13.3%)	5 (5.0%)

The analysis found no statistically significant association between sex and the development of post-hepaticojejunostomy complications. Although a higher proportion of females were affected by both cholangitis and hepaticojejunostomy strictures, the chi-square tests yielded p-values of 0.53 and 0.58, respectively, indicating that these observed differences were not statistically significant. The inference was that sex alone was not a significant independent risk factor for these complications. The higher crude rates observed in females are likely attributable to other confounding factors, such as the underlying disease distribution, rather than a biological predisposition linked to sex (Table 3).

**Table 3:** Association of sex with cholangitis and hepaticojejunostomy stricture. Values are expressed as a number (percentage within sex); chi-square test was used for categorical comparisons. p > 0.05 was considered not statistically significant.

Variable	Total (n)	Males (n = 34)	Females (n = 66)	χ²	p-value
Cholangitis	39	9 (26.5%)	30 (45.5%)	0.39	0.53
Hepaticojejunostomy stricture	20	3 (8.8%)	17 (25.8%)	0.32	0.58

The analysis revealed no statistically significant association between age and the development of cholangitis (p = 0.643). Although this association did not reach statistical significance (p = 0.063), it was interpreted as an observed trend and not as a definitive risk factor. The incidence of stricture was highest in the younger cohort and notably lower in patients over 40 years of age. This pattern suggests that younger age may be a potential risk factor for stricture formation post-surgery, possibly related to different healing responses or disease etiology. Although not definitive, this trend indicates that younger patients might benefit from more vigilant postoperative surveillance for this complication (Table 4).

**Table 4:** Distribution of cholangitis and hepaticojejunostomy stricture by age group. Percentages were calculated with respect to total cases of each complication; values are expressed as a number (percentage); the chi-square test was applied across age groups. p > 0.05 was considered not statistically significant.

Age group (years)	N	Cholangitis (n = 39)	χ²	p-value	Hepaticojejunostomy stricture (n = 20)	χ²	p-value
10-20	9	4 (10.3%)	1.54	0.643	4 (20.0%)	7.30	0.063
21-30	27	12 (30.8%)			7 (35.0%)		
31-40	34	14 (35.9%)			7 (35.0%)		
>40	30	9 (23.1%)			2 (10.0%)		

The analysis identified two postoperative factors that were strongly associated with hepaticojejunostomy stricture formation. The presence of a postoperative bile leak significantly increased the stricture rate to 6 (42.9%) compared to 14 (16.3%) without a leak (p = 0.021). Furthermore, postoperative cholangitis was a critical risk factor, with 18 (50%) affected patients developing a stricture versus only 2 (3.1%) without cholangitis (p < 0.001). The inference is that postoperative bile leak and cholangitis are major modifiable drivers of anastomotic strictures. This underscores the paramount importance of meticulous surgical techniques to prevent leaks and aggressive early management of cholangitis to mitigate the risk of serious long-term complications (Table 5).

**Table 5:** Postoperative factors associated with hepaticojejunostomy stricture formation. Values are expressed as numbers (percentage within row); chi-square test was used for comparison. *Statistically significant (p < 0.05).

Postoperative factor		Hepaticojejunostomy stricture present (n = 20)	Hepaticojejunostomy stricture absent (n = 80)	χ²	p-value
Postoperative bile leak	Present	6 (42.9%)	8 (57.1%)	5.32	0.021*
	Absent	14 (16.3%)	72 (83.7%)		
Postoperative cholangitis	Present	18 (50.0%)	18 (50.0%)	31.64	0.001*
	Absent	2 (3.1%)	62 (96.9%)		

## Discussion

The present study evaluated the long-term outcomes of 100 patients who underwent Roux-en-Y hepaticojejunostomy for benign biliary diseases, revealing a 20% incidence of anastomotic stricture and 39% incidence of recurrent cholangitis over a median follow-up exceeding six months. These rates align with the existing literature, where stricture incidence ranges from 10-25% in large cohorts [3,4,9]. For instance, a systematic review by Halle-Smith et al. reported a pooled stricture rate of 18% after hepaticojejunostomy for bile duct injuries, attributing the variability to surgical expertise and patient selection [4]. Similarly, cholangitis rates of 20-40% have been documented in studies on benign biliary reconstruction, often linked to bacterial ascension via the Roux limb [3,10]. The higher complication burden in our cohort may stem from the inclusion of diverse etiologies, including oriental cholangiohepatitis, which predisposes patients to recurrent infections owing to intrahepatic stones and stasis. In specialized centers, improved short-term outcomes (near-zero mortality) reflect advancements in perioperative care, yet long-term morbidity persists, underscoring the need for refined techniques, such as the Hepp-Couinaud approach, to minimize tension and ischemia at the anastomosis [11].

Complication rates varied significantly by surgical indication, with patients with bile duct injury experiencing the highest incidence of stricture (higher than choledochal cysts or other groups). This finding is supported by multiple studies identifying bile duct injury as a key risk factor for adverse outcomes [3,4]. The underlying mechanism likely involves iatrogenic trauma leading to vascular compromise and fibrosis. For example, concomitant arterial injuries during cholecystectomy can cause ischemic strictures. A previous study reported that anastomotic stricture is influenced by choledochal cysts, symptom duration, and prior inflammation [12]. Our "other" group, including strictures from cholangiohepatitis, showed intermediate risks. Oto et al. assessed the long-term effects and quality of life following bile duct injury repair with a mean follow-up period of 150 months. They reported that 78.6% of the patients remained symptom-free for more than five years [13].

Our observed anastomotic stricture rate (20%) is higher than many large series reporting rates in the 8-12% range. Several factors likely explain this difference. First, case-mix: one-third of our cohort had post-cholecystectomy bile duct injuries, which are known to lead to a substantially higher risk of stricture after reconstruction. Second, referral and selection bias at tertiary centers may lead to enrichment in the management of complex injuries and difficult reconstructions. Third, follow-up and surveillance practices influence detection: routine use of MRCP and active case-finding increase the identification of asymptomatic or subclinical strictures that may be missed in series relying on symptom-driven follow-up. Finally, attrition of asymptomatic patients in real-world Indian practice (who often do not return for routine visits) may paradoxically enrich our follow-up cohort for symptomatic patients, increasing the apparent complication proportion. 

Despite the female predominance (66%), no significant association was found between sex and complications. This echoes prior research, where sex has not emerged as an independent predictor, and a large series by Cho et al. reported no sex differences in hepaticojejunostomy outcomes, suggesting that observed disparities arise from disease epidemiology rather than biological factors, such as hormonal influences on healing [14]. Confounding by etiology likely explains our higher crude rates in the female population, as bile duct injury and cysts disproportionately affect women.

Bansal et al. assessed predictors influencing long-term outcomes in hepaticojejunostomy by utilizing the Terblanche grading system. Their univariate analysis revealed six factors correlated with unfavorable long-term results: a history of laparoscopic cholecystectomy, a higher degree of injury, an extended duration of referral from the initial surgery, previous attempts at repair, and concurrent vascular injury [15]. Nevertheless, only the degree of injury demonstrated an independent association with overall adverse outcomes in the multivariate analysis.

A near-significant trend linked younger age (<40 years) to higher stricture rates with no age effect on cholangitis. Dimou et al. [9] reported that the incidence of strictures is lower in younger patients. This aligns with the findings of Chen et al., who identified younger age as a risk factor for biliary strictures post-reconstruction, possibly due to more vigorous fibrotic responses in young tissues or aggressive disease presentations in pediatric-onset conditions such as choledochal cysts [16]. Older patients may benefit from reduced inflammatory activity, although comorbidities could be confounded, and our exclusion of major comorbidities strengthens this observation. Mechanistically, younger age correlates with prolonged survival and thus, greater cumulative exposure to recurrent insults.

Postoperative bile leak and cholangitis have emerged as strong predictors of strictures. Bile leaks, which occurred in 14% of our cases, promote perianastomotic inflammation and fibrosis. In a large retrospective study of 583 patients, Okabayashi et al. reported a postoperative cholangitis incidence of 7.7% after biliary-enteric anastomosis, with anastomotic stricture developing in 57.8% of affected cases and managed successfully by non-operative intervention. Early leaks may indicate technical issues such as suture tension or poor vascularity, whereas cholangitis often follows stasis or incomplete stone clearance [8]. These findings emphasize the prevention of leaks through meticulous anastomosis and aggressive management of infections with antibiotics and drainage.

Clinical implications

Our results advocate for risk-stratified follow-up: intensive surveillance (such as quarterly imaging and labs) for patients with bile duct injury, younger individuals, and those with early complications to enable timely interventions such as percutaneous dilatation. Prophylactic measures, such as access loops in high-risk cases, could reduce reoperations, improve quality of life, and prevent cirrhosis.

Limitations

This study has several limitations. Its retrospective design is inherently subject to selection and information bias. As a tertiary referral-center experience, the cohort may be enriched for more complex biliary pathology, particularly bile duct injury, potentially limiting generalizability. Follow-up duration, while sufficient to capture most early and intermediate strictures, was variable, and very late complications occurring years after surgery may not have been detected. In addition, in routine clinical practice within the Indian healthcare setting, asymptomatic patients or those who relocate frequently do not return for prolonged surveillance. This loss to follow-up may result in overrepresentation of symptomatic patients, thereby inflating the observed complication rates. Technique-specific analyses, including the impact of timing of bile duct injury repair or the use of the Hepp-Couinaud approach, were not feasible due to non-randomized operative decision-making, uneven subgroup sizes, and limited statistical power, raising the risk of selection bias. Finally, although several associations were identified, causal inferences cannot be drawn, and some findings should be regarded as hypothesis-generating, warranting confirmation in prospective, multicenter studies with standardized long-term follow-up.

## Conclusions

Roux-en-Y hepaticojejunostomy for benign biliary disease is associated with substantial late morbidity, particularly in patients undergoing reconstruction for bile duct injury. Patients with BDI, especially those who develop postoperative bile leaks or early cholangitis, represent a high-risk subgroup and should undergo structured radiological surveillance with MRCP for a minimum of 24 months, even in the absence of symptoms, to enable early detection of anastomotic narrowing. Prevention of bile leak through meticulous construction of a wide, tension-free mucosa-to-mucosa anastomosis and aggressive early management of cholangitis should be considered the primary surgical targets to improve long-term success. Younger patients may warrant closer follow-up given their longer lifetime risk. Adoption of risk-stratified, protocol-based surveillance strategies has the potential to reduce progression to hepatolithiasis, liver abscesses, and secondary biliary cirrhosis, thereby preserving long-term quality of life.
